# Supplementary material for: Long-Chain Modification of the Tips and Inner Walls of MWCNTs and Their Nanocomposite Reverse Osmosis Membranes
Source: Membranes (Basel). 2022 Aug 18;12(8):794. doi: 10.3390/membranes12080794 (PMC9415691; doi:10.3390/membranes12080794)
Supplement: Supplementary file 1 [file membranes-12-00794-s001.zip › membranes-1829599-supplementary.pdf]

membranes

Supplementary Materials

Long-Chain Modification of the Tips and Inner Walls of MWCNTs and Their Nanocomposite Reverse Osmosis Membranes

Qing Li [1], Dengfeng Yang [2], Qingzhi Liu [2], Jianhua Wang [2,*], Zhun Ma [1], Dongmei Xu [1] and Jun Gao [1]

[1] College of Chemical and Biological Engineering, Shandong University of Science and Technology, Qingdao 266590, China
[2] College of Chemistry and Pharmaceutical Science, Qingdao Agriculture University, Qingdao 266109, China
* Correspondence: ygdbbwjh@163.com.

Table S1. Materials used in experiment.

Materials	Specification	Sources
MWCNTs	Purity 98%(Raman), diameter 4.9 nm, Length: 10 to 30 um, Ash<0.5wt%, SSA>350m2/g, EC>100s/cm	Beijing gold deco island technology Co., Ltd
Sulfuric acid	Guaranteed reagent	Laiyang Kant Chemical Co., LTD
hydrochloric acid	Guaranteed reagent	Laiyang Kant Chemical Co., LTD
nitric acid	Guaranteed reagent	Laiyang Kant Chemical Co., LTD
dimethylformamide (DMF)	Analytically pure	Tianjin Yongda Chemical Reagent Co. LTD
lithium aluminum hydride (LiAlH4)	Analytically pure	Shanghai Maclean Biochemical Technology Co., LTD
pyridine	Analytically pure	Tianjin Yongda Chemical Reagent Co. LTD
tetrahydrofuran	Analytically pure	Tianjin Fuyu Fine Chemical Co. LTD
ethyl acetate	Analytically pure	
triethylamine	Analytically pure	Aladdin Chemical Co., LTD
dimethyl sulfoxide	Analytically pure	Sinopharm Group Chemical Reagent Co. LTD
m-phenylenediamine (MPD)	Analytically pure	Tianjin Beilian Fine Chemicals Co. LTD
dodecanedioic acid		Shanghai Maclean Biochemical Technology Co., LTD
trimesoyl chloride(TMC)	Analytically pure	Shanghai Maclean Biochemical Technology Co., LTD
camphor-10-sulfonic acid	Analytically pure	Aladdin Chemical Co., LTD
sodium dodecyl sulfate	Chemically pure	Tianjin Bodi Chemical Co., LTD
toluene	Analytically pure	Laiyang Kant Chemical Co., LTD
oxalyl chloride	Chemically pure	Chengdu Kelong Chemical Reagent Factory

sodium hydrogen carbonate (NaHCO3)	Analytically pure	Tianjin BASF Chemical Co., LTD
sodium chloride	Analytically pure	Tianjin BASF Chemical Co., LTD
anhydrous magnesium sulfate	Analytically pure	Tianjin BASF Chemical Co., LTD
potassium hydroxide	Analytically pure	Tianjin BASF Chemical Co., LTD
methanol	Analytically pure	Tianjin Fuchen Chemical Reagent Factory
ethanol	Analytically pure	Tianjin Fuchen Chemical Reagent Factory
n-hexane	Analytically pure	Sinopharm Group Chemical Reagent Co. LTD
polysulfone (PSF) membranes	Aperture: 0.04 0.22 (microns)	Shenzhen Jiachun Membrane Filtration Equipment Co. LTD
polytetrafluoroethylene (PTFE) membranes	Diameter 50mm, aperture 0.22um	Haiyan New Oriental Plasticizing Technology Co. LTD

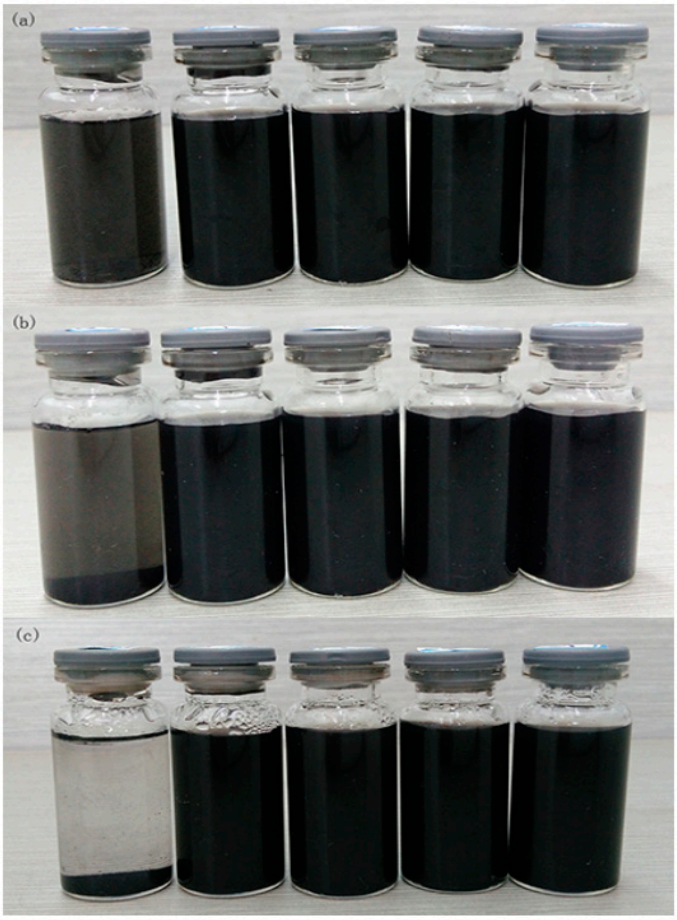

Figure S1. Dispersion and stability of samples after ultrasonic 0 h (**a**), 1 h (**b**), 40 weeks (**c**), (samples from left to right are MWCNT, MWCNT-COOH, MWCNT-CH_2OH, MWCNT-COCl , MWCNT-$CH_2OCOC_{12}H_{23}O_3$).

In order to determine the effect of chemical modification on the dispersibility and stability of carbon nanotubes, the water dispersibility of MWCNTs was studied by static sedimentation method. MWCNT, MWCNT-COOH, MWCNT-CH_2OH, MWCNT-COCl , MWCNT-$CH_2OCOC_{12}H_{23}O_3$ was dispersed in water, sonicated for 30 min, and allowed to stand for a while. Figure 2-12 lists the water dispersion of the samples after 0 h (a), 1 h (b), and 40 weeks (c) of ultrasonic standing (samples from left to right are MWCNT, MWCNT-COOH, MWCNT- CH_2OH, MWCNT-COCl, MWCNT-$CH_2OCOC_{12}H_{23}O_3$). As shown in Figure S1, the MWCNTs began to coagulate and precipitate after 1 hour of ultrasonic standing, while the MWCNTs with grafted functional groups still maintained good dispersibility and stability after ultrasonic storage for 40 weeks or even longer.

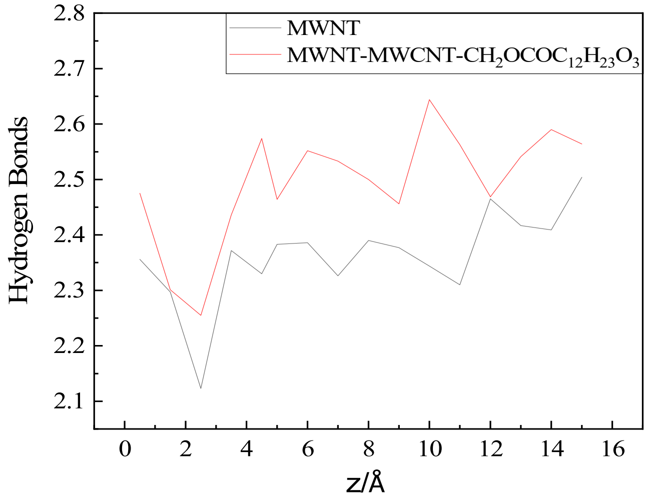

Figure S2. Hydrogen Bonds of water molecules in (**a**) MNNTs (**b**) MWCNT-$CH_2OCOC_{12}H_{23}O_3$.
